# Challenges of and corrective recommendations for healthcare-associated infection’s case findings and reporting from local to national level in Iran: a qualitative study

**DOI:** 10.1186/s12912-022-00976-1

**Published:** 2022-07-19

**Authors:** Nahid Dehghan-Nayeri, Arash Seifi, Leili Rostamnia, Shokoh Varaei, Vahid Ghanbari, Ali Akbari Sari, Hamid Haghani

**Affiliations:** 1 Nursing and Midwifery care research center, School of Nursing and Midwifery, Tehran University of Medical Sciences, Tehran, Iran; 2 Department of Infectious Diseases, Faculty of medicine, Tehran University of Medical Sciences, Tehran, Iran; 3 Nursing Department, School of Nursing and Midwifery, Kermanshah University of Medical Sciences, Esar square, Kermanshah, Iran; 4 Medical-Surgical Department, School of Nursing and Midwifery, Tehran University of Medical Sciences, Tehran, Iran; 5 Nursing Department, School of Nursing and Midwifery, Kermanshah University of Medical Sciences, Kermanshah, Iran; 6 Department of Health Management and Economics, School of Public Health, Tehran University of Medical Sciences, Tehran, Iran; 7 College of Management and Medical Information, Iran University of Medical Sciences, Tehran, Iran

**Keywords:** Health-care associated infection, Surveillance, Case-finding, Reporting, Qualitative study

## Abstract

**Background:**

The accuracy of health care−associated infections (HAIs) statistics in many countries is questionable and the main reasons of this inaccuracy are not well-known. The study aim was to explore inhibitors of and corrective recommendations for HAIs case findings and reporting in some of Iran hospitals.

**Methods:**

Sixteen face-to-face interviews and an expert panel were performed with expertise of infection prevention and control (IPC) programs in hospitals, and Deputies of Health and Treatment in medical university and Ministry of Health from Feb 2018 to May 2019. Using conventional content analysis, code, subcategories and categories were developed.

**Result:**

Three categories emerged including improper structure preparation, conflict of interest, and inadequate motivation. Allocating distinct budget and adequate staff to IPC programs, developing a user-friendly surveillance system and engaging physicians and nurses for HAIs reporting are the main corrective recommendations accepted by the expert panel.

**Conclusion:**

Despite the improvement in growing case-findings and reporting of HAIs in Iran, there are many challenges which inhibit accurate case finding and reporting of HAIs. So it is necessary to update the structure, system and rules to reach accurate HAIs data in Iran.

## Background

Health care−associated infections (HAIs) are increasing the patient safety concerns worldwide [1, 2]. HAIs are associated with increased length of hospitalization, morbidity, mortality and economic burden. For these reasons infection prevention and control (IPC) programs have become a major priority for health-care organizations in most countries [3]. Nosocomial Infection Surveillance System (NISS), as a part of IPC program, was established in Iran in 2007 [4].

According to a national report in 2015, the rate of HAIs was 1.18% in Iranian hospitals [5]. Seifi et al., in 2019, showed that the sensitivity of HAIs reporting in Intensive care units is less than 30% [4]. Kousha et al., and Esfandiari et al., in their studies well-founded that under-reporting is one of the important challenges in the IPC program in Iran [6, 7]. Pezhman et al., in their study with aim report the status of NIs and to evaluate the Iranian nosocomial infection surveillance system (INISS) in a teaching hospital in the south of Iran indicated the rate of HAIs reporting was low [8]. Other investigations which were conducted in other countries also indicated that under and over-reporting of HAIs is one of the greatest challenges of HAIs reporting [4, 9–14]. This inaccuracy in HAIs reporting could lead to a lack of or incomplete treatment of infectious patients, reduction of quality of patient care, the transmission of HAIs to other members of the community, wrong decisions and policy-making [6, 10, 11, 15]. So, accurate data are a critical aspect of successful implementation of IPC programs.

Previous works in this field suggested that multitasking of infection control nurses (ICNs), an inadequate knowledge and preparation of ICNs for case finding, an inadequate collaboration of physicians and nurses in case finding and reporting, and inactivation of infection control link nurses are the main barriers to accurate HAIs reporting [1, 4, 6, 16, 17].

A key problem with much of the literatures on HAIs surveillance system is that most of them look quantitatively at HAIs reporting. Therefore, considerable ambiguity exists with regard to the main causes of HAIs data inaccuracy.

The aim of this study was to broaden the current knowledge of HAIs reporting challenges from hospitals to the Ministry of Health (MOH) and to discover innovative corrective recommendations to deal with emerging challenges. The results of this study would be valuable for policymakers to improve the current IPC programs.

## Methods

A two-phase qualitative study (conventional content analysis and expert panel) was done from Feb 2018 to May 2019. The first phase was face-to-face interviews conducted to explore the HAIs challenges in Iran. Then, an expert panel was held to provide corrective recommendations in order to tackle the extracted challenges in the previous stage.

### Participants

Participants included infection control nurses, physicians from hospitals and technical officers affiliated with Tehran University of Medical Sciences (TUMS) and MOH infection control committee members. The participants of two phases were chosen using purposeful sampling methods.

The expert panel included eight people who have clinical, managerial, research, and policy-making experiences regarding IPC programs in educational hospitals and in health, and treatment deputies of TUMS and MOH. Everyone who was invited to participate in the study accepted our invitation.

In both phases of the study, inclusion criteria were having at least 1 year of experience in their current position related to IPC programs in hospital, treatment deputies of TUMS and MOH. Being willing to participate in the study was another inclusion criterion. Exclusion criteria include anyone unwilling to participate.

### Data collection

Face to face in-depth, semi-structured, and audio-recorded interviews were conducted in the participants’ workplace. At the beginning of the interviews, participants were asked to fill out a self-completion demographic questionnaire and sign an informed consent form. The interviews were performed by the corresponding author and lasted 45-100 minutes. The focus of the interviews was on participants’ experiences and opinions regarding HAIs reporting challenges and their recommendations to resolve these challenges. A guide for interviews including research aims and the interview questions was developed by the research team and piloted in three interviews. The main questions of the interview were*: Based on your experiences in the IPC program*, *how is the HAIs reporting process? Would you please share with me your experiences with the HIAs case finding and reporting process? What are your suggestions or actions have you taken to improve reporting and case finding?* The open-ended questions were followed by probing questions. Data gathering and analysis were conducted simultaneously. Sampling and interviews were stopped when the categories were explored and data saturation was achieved. After 16 interviews, no new code was generated from the interviews data.

The expert panel meeting was held in one of the educational hospitals of TUMS in May 2019. The session lasted 4 h. First, the key results of the previous stage and the meeting objectives were presented to participants by the first author. In addition, each participant was given a copy of the presented results. Then, they were given an hour to write their recommendations for solving each of the challenges that were extracted in the interviews regarding HIAs reporting in Iran. Finally, recommendations for each of the challenges were presented by the participants in the interviews, and expert panel members were discussed regarding the practicability of implementation. Eventually, the recommendations were accepted by more than half of the participants were recorded as a corrective recommendations for each HAIs reporting challenge.

### Data analysis

A conventional content analysis was employed to analyze the data that were gathered through interviews. This method is commonly used to gain an in-depth understanding of health policies and plans [18]. Interviews were transcribed verbatim. The interviews text was read several times thoroughly by two of the researchers (L.R. and V.GH.) to gain a general understanding of the data. After that, meaning units were extracted from the text and condensed. Then, the condensed meaning units were abstracted and labeled as codes. Finally, sub-categories and categories were developed based on a constant comparison of codes regarding their content similarities and differences [18]. MAXQDA 2010 software (VERBI Software GmbH) was used for the management and analysis of the data.

The corrective recommendations which were suggested by the expert panel were also categorized into three categories (*hospital, medical university, and MOH)* to determine which department or organization would be in charge of implementing each recommendation.

### Trustworthiness

Credibility, dependability, and transferability were suggested to ensure the trustworthiness of content analysis research [18]. Prolonged engagement (the research process lasted about 15 months) and data collection methods triangulation (interviews, document reviews, and expert panel), and maximum variation of the participants, boost the credibility of the findings. Cross and peer-check were used to enhance the dependability and confirmability of the findings. The external reviewers who are working in the health system and are skilled in qualitative research confirmed the process and content of data analysis. Clear and distinct descriptions of the research process and data analysis were applied to increase finding transferability.

### Ethical consideration

Research Ethics Committee of Nursing and Midwifery Faculty of Tehran University of medical sciences approved the research project and supervised it (Registration number: IR.TUMS FNM.REC.1396.3212). Participants were informed that their participation in the research project is voluntary and they could refuse to participate or withdraw from the project at any time. Furthermore, participants signed a written consent form. Lastly, participants were reassured that their information would be confidential. All methods were carried out in accordance with relevant guidelines and regulations of qualitative research.

## Result

Participants included 12 women and four men. The mean age of participants was 44.94 ± 7.39. Job experience of participants in a recent post related to IPC in the study ranged from 1to 23 years with a mean of 8.19 ± 6.34 years. Participants’ job characteristics are shown in Table 1. Analysis of the data resulted in three main categories: Improper structure preparation; conflict of interest; inadequate motivation (Table 2). The following section outlines the participants’ views and experiences related to HAIs reporting challenges in Iran. The corrective recommendations which were suggested in the expert panel are presented in Table 3.

**Table 1:** Individual characteristics of participants in the study of INIS reporting challenges and corrective recommendations

Row	Organization level	Job position	Job experience
1	MoH-Food and Drug Organization	High level manager	5
2	MoH-Health Deputy	Technical officer in HAIs prevention and control	13
3	MoH-Deputy of Curative Affaires	Technical officer in charge of patient safety	23
4	MOH	Senior Assessor of the Ministry of health	10
5	MoH, Medical University, hospital	Member of infection control committee (the National, university, and hospital)	21
6	Medical University	Technical officer in deputy of medical affaires	2
7	Medical University	Insurance, Tariff & Standard Coordinator Expert	8
8	Hospitals	Head of infectious diseases ward	7
9	Hospitals	Head of infectious diseases ward	5
10	Hospitals	Infection control nurse	3
11	Hospitals	Infection control nurse	3
12	Hospitals	Infection control nurse	10
13	Hospitals	Infection control nurse	1
14	Hospitals	Infection control nurse	10
15	Hospitals	Infection control nurse	6
16	Hospitals	Infection control nurse	4

**Table 2:** Codes, subcategories, and categories of challenges with reporting of Healthcare-Associated Infections in Iran

Categories	Subcategories	Codes
Improper structure preparation	Weaknesses in the HAIs recording system and following up with patient	Lack of HAIs fallow up system in discharged patients
		Lack of a data collection system from the clinics
		Lack of access to information about the infectious patients when referring to other treatment centers
		Failure to record information of infectious patient referred to physicians’ office
		Lack of an integrated patient information system (electronic patient records)
		Statistics on healthcare-related infections are based solely on hospital information
	Shortage of human resources	Incompatibility of the number of ICNs with hospital beds
		Lack of human resources to fallowing up
		Multi-tasking of ICNs
	Insufficient activity regulations for ICLNs	ICLNs activities upon request and coercion
		Acting by relation, not by responsibility
		Inactivation of ICLNs
		Improper performance of ICLNs
		Multitasking of ICLNs
	Infrastructure and budgetary problems	Most labs fail to provide reliable data
		Insufficient funding for infection control unit by MOH
		Non-allocation of separate funds for the activities of ICLNs
		Lack of funding for the development of Iranian nosocomial surveillance system software
Conflict of interest	Fear of compromising interests	Fear of losing clients
		Concern about the organization’s follow-up and its consequences
		Fear of negative reputation
		Worry about taking action against yourself
		Fear of diminishing benefits
	Hidden pressure	Indirect targeting of MOH / Hospitals to overreporting of HAIs
		Over reporting with good reporting motivation
		Exaggeration in HAIs reporting due to the incentive to get a reward from the university
		Anxiety caused by a low rate of HAIs
Inadequate motivation	High workload	Multi-tasking of Infection Control Physician
		Nurses’ unwillingness to HAIs reporting due to high workload
		Unwillingness to accept the post of ICN because of high workload
	Poor quality of training and educational programs	Insufficient training at the beginning of their work (as an ICN/ICLN) - Inadequate preparation of ICN to take responsibility - Self-study about infection control at the beginning of responsibility - Lack of a plan to prepare the nurse for ICN or ICLN
	No financial incentive	Lack of insufficient funding for ICN
		Get little for performing tasks
		The job of infection control practitioners is almost free
		Incompatibility of work and income

*Abbreviations*: *ICN* Infection control nurse, *ICLN* Infection control link nurse, *MOH* Ministry of health

**Table 3:** Corrective recommendations to deal with challenges of HAIs reporting in Iran

Challenges	Corrective recommendations	In charge
Problems with case finding and recording of HAIs	➢ Motivating and engaging physicians for reporting of HAIs cases from their office or clinic	MOH
	➢ Providing required tools to register HAIs cases in INIS	
	➢ Collaboration of the follow-up unit and ICNs	
Budgetary and infrastructure	➢ Allocating separate funds to the Infection Control Unit in order to carry out educational, research, implementation, and development activities of IPC programs.	
	➢ Correcting resource allocation to physicians, nurses, and IPC programs.	
	➢ Providing laboratory infrastructures such as PCR for antibiotic resistance detection and confirmation of microorganisms	
Hidden pressure	➢ Evaluation of the case finding and reporting process instead of evaluating the reported results and figures	
	➢ Correct targeting for reporting	
	➢ Justification of various hospital/university and departmental authorities regarding the purpose of case finding through training courses	
Insufficient activity regulations for ICLNs	➢ Clarifying, writing, and approving job descriptions of infection control practitioners	
	➢ Motivate the ICLNs through incentive payments and certificates that are effective in annually evaluating and promoting customers	
Poor quality of training and educational programs	➢ Training case finding, reporting, and IPC management while studying at university (for various disciplines)	University of Medical Sciences
	➢ Developing master’s degree program in infection prevention and control	
	➢ Justifying managers to welcome the course and support a trained nurse	
	➢ Development of short-term in-service training programs for different hospital categories (therapeutic, non-therapeutic), continuous and follow-up courses	
	➢ Annual job promotion is subject to training courses	
	➢ Provide real and virtual educational media to educate community-based infection control patients, patients, and caregivers	
No financial incentive	➢ Performance-based payment to Physician of infection control, ICNs and ICLNs	
Fear of compromising interests	➢ Clarification of the contribution of each part (system, staff, patients) in HAIs	Hospitals
	➢ Verifying HAIs cases by the physician of the IPC committee	
	➢ Developing laws and overseeing the proper implementation of the rules regarding surgical indication	
	➢ Pay attention to the performance of the treatment team (physician, nurse, etc.) associated with infection prevention and control programs in financial payments	
	➢ Providing feedback to surgeons regarding their performance in the field of IPC	
	➢ Administrative encouragement and punishment by presenting commendation plates or written notes regarding compliance with infection prevention and control principles during treatment and care of patients.	
High workload and Shortage of human resources	➢ Choosing the right criteria for selecting an infection control expert	
	- Compilation and standardization of the calculation of the number of infection control nurses in each hospital (per hospital beds, per ICU beds, per high-risk patients, per high-risk ward)	
	➢ Implementing a full-time infection control nurse in hospitals	
	➢ Removing multiple tasks unrelated to infection control from nurse assigned infection control tasks	
	➢ Adequate staff allocation to implement and track the affairs of infection control programs	
	➢ Pay attention to the duties of ICLNs while shifting the duties of the nurse staff by the nurse in charge of the shift.	

*Abbreviations*: *HAIs* Healthcare-Associated Infections, *IPC* Infection Prevention and Control, *MOH* Ministry of health, *ICLN* Infection control link nurse

### Improper structure preparation

Nearly half of the participants expressed that accurate HAIs reporting needs adequate and appropriate resources. These resources including HAIs registry, human resources, financial support, and laboratory diagnostic equipment were expressed by participants as common causes of under-reporting of HAIs in the Iranian nosocomial infections surveillance system (INIS).

All of the ICNs and doctors of IPC indicated that no system and program exist for tracking and recording of HAIs cases among discharged patients. They stated that although the INIS has been established to register HAIs information, only HAIs which are detected in hospital wards could be reported in the INIS system. It is impossible to report HAIs cases which are detected in clinics or physician offices in the INIS system. Moreover, discharged patients have not followed regarding HAIs probably symptoms. So, it is possible a large number of patients who have signs and symptoms of HAIs and go to offices and clinics are not detected and recorded. Then, they believed it is necessary to improve the INIS in order to be able to register HAIs cases from other health care facilities. An IPC physician said *“Some cases of HIAs are not detected because some surgeons treat these patients in their offices or clinics. So, if we are able to document these HAIs cases, real statistics of HAIs would be possible (participant #6).*

Moreover, one of the ICNs stated that *“It is possible that patients who are discharged from the hospitals show their HAIs signs and symptoms at home. Then, following patients in this respect, could lead to detection and reporting of these HAIs cases” (participant #2).*

Performing IPCs programs especially HAIs case findings and reporting needs sufficient human resources. It was stated that each hospital has an ICN. Moreover, in some hospitals, other tasks have been delegated to an ICN. So, ICNs do not have enough time to visit wards and check patients’ documents or laboratory tests or follow-up discharged patients. Some of the participants believe that the workload of ICNs was not estimated properly at the beginning of implementing IPC programs in Iranian hospitals. “*In some hospitals, there are 500-600 beds with various patients (immunodeficiency or transplanted patients or resistant infections) and just an ICN, besides other IPCs programs, should check all of the patients’ documents for HAIs case finding. So it seems impossible for an ICN to do all of these tasks simultaneously” (participant #11).*

Interviewees’ experience indicated that IPC programs do not have adequate funding. Participants noted that no funds are allocated for Infection Control Link Nurses (ICLNs) activities. Even, no funding is allocated for the development or updating of the registry system of HAIs reporting infections. One IPC physician said “*Developed countries even have separate budgets for developing or updating a surveillance system, while in our country there is no such thing at all. We’ve developed INIS and updated it two times, without any funding or paying for it” (Participant # 6).*

Nearly all participants who work in hospitals mentioned that diagnosis of some HAIs cases requires up-to-date laboratories tools and kits, it is stated that many labs do not have access to such equipment. So this can lead to poor detection of HAIs in blood, sputum, or other body fluids. On the other side, some participants also reported that the ICNs did not have access to the results of patient culture samples through the Hospital Information System (HIS).

### Conflict of interest

Some participants stated that for a variety of reasons such as fear of a salary cut or losing clients and worries about a negative reputation, some physicians may be reluctant to report HAI cases.

One of the ICNs expressed:

“*One of our doctors said that, if the head of the hospital saw the number of my patients with related HAIs that you have reported, certainly he would reduce my salary” (Participant #8).*

In addition to reluctance of physicians to report HAIs cases, some participants believed that a high number of HAIs could negatively influence hospitals in respect of reputation, financially and legally. They stated if a hospital is known for a high number of HAIs, it might lessen the number of customers and hospital administrators would be legally responsible to the court.

#### A member of the National Committee of IPC noted

*“For example, if a hospital becomes known for its high infection rate, the number of customers will probably decrease or when some patients’ documents are referred to forensic medicine with regard to assessing the role of the physician, it could lead to a bad reputation for both the physician and the hospital. So it is clear that they are reluctant to report HAIs in their patients” (Participant #11).*

Surprisingly, nearly half of the participants mentioned that recent changes in national policies to promote HAIs reporting have created a tendency among hospitals to achieve a higher benefit by reporting HAIs. They expressed that in recent changes, a hospital with a higher number of HAIs, was introduced as the best hospital regarding case finding and reporting HAIs by MOH. So, a view has been formed among some hospitals managers that over-reporting is better than underreporting. Therefore, they may like ICNs to report a higher rate of HAIs. Then, if an ICN reports a few numbers of HAIs, s/he may be worried about acceptance of her/his report. An ICN described her experience in this regard:

*“There is pressure on us to report more HAIs. For example, if I do not have a patient with HAIs in an intensive care unit (ICU) for a month, I become anxious! Then I go and asked the nurses: We did not have any HAIs? If I send zero number of HAIs for ICU to the health center, the accuracy of the report will come into question. This may be a factor for overreporting “(Participant# 9).*

### Inadequate motivation

This category is related to issues that influence IPC team members’ performance. The participants claimed that delegating several tasks to an ICN or an IPC doctor leads to them becoming very busy. Moreover, the other members of a healthcare team, especially nurses, are unwilling to collaborate with ICNs for HAIs detection and reporting. Some participants noted that these problems led to a nurse or a physician having no interest in becoming a member of the IPC team.

An ICN expressed:

*IPC tasks are numerous. I should assess patients’ medical records, audit surgical antibiotic prophylaxis, vancomycin serum level, and offer infection prevention solutions, do tasks related to IPC unit accreditation, do official follow-up and coordination, and ... (Participant #8).*

Nearly all of the ICNs and IPC doctors pointed out that all members of the IPC team should be trained on how to carry out IPC programs such as HAIs detection and reporting. They underlined that ICNs and IPC doctors do not receive any specialized training for this position. Moreover, most of the ICNs declared that they became familiar with the IPC program via self-study or by consulting with other hospital ICNs.

One of the ICNs said, *“When I became an ICN, my previous colleague taught me something, I read the MOH guideline book, or I had searched for a series of things on the Internet” (Participant #8).*

The study participants stated many examples, which indicated there is no monetary incentive for IPC members. Most of the ICNs and the IPC doctors claimed that despite their responsibilities and tasks in the IPC committee, they do not receive any extra payment.

One of the IPC doctors expressed:

*All IPC doctors in Iran work for free. We attend sessions and do a thousand other tasks like checking laboratory reports, and protocol writing, while we have no financial gain, so no interest remains in working (Participant #11).*

It is also mentioned that an ICLN in each hospital ward has been selected to help the ICN to implement IPC programs. According to the ICNs expressions, the ICLN has been asked to collaborate with ICN without any change to their routine nursing tasks or any monetary motivation. Although it is possible in some cases for ICLNs to do activities because of their individual interests or personal relations with ICNs, most ICLNs are inactive and have no contribution to the implementation of IPCs programs in their wards.

“*The ICLN should do their IPC task in addition to her/his routine nursing tasks, therefore, they are inactive. They will be more active if they are paid extra for their related activities “(Participant #2).*

## Discussion

The present study explored some of the contributing factors to inaccuracy reporting of HAIs and corrective recommendations to deal with them from the ICNs and ICPs and other stakeholders’ perspectives in Iran. The finding revealed improper structural preparation, conflict of interest and inadequate motivation of members of the IPC team are leading causes of HAIs under or over-reporting.

The lack of a comprehensive and integrated system to record HAIs cases from hospitals, clinics, and physician offices and the lack of patient follow-up lead to underreporting of HAIs. This finding is consistent with Rodríguez et al., which expressed that access to electronic records of patients is one of the necessities to perform IPC programs [9]. As reported by Kousha et al., lack of follow-up patients is one of the reasons for underreporting of HAIs [6]. More recent studies in Iran revealed that a follow-up patient system is required to reach accurate information about HAIs prevalence [7, 19]. Gia To et al. and Rosenthal show that performing a follow-up system could significantly increase the number of HAIs cases among discharged surgical patients [10, 11]. To manage this problem, the experts in the panel recommended developing a comprehensive and user-friendly system for reporting of HAIs cases from other parts (such as clinics, and physician offices) and encouraging physicians to report HAI cases from clinics and their offices. It is also recommended the follow-up care unit should have close collaboration with the IPC unit in order to find HAIs cases in other health care services. Therefore, having an integrated system that can collect data from different parts of the health system (hospitals, offices, clinics ...) will provide access to more accurate information about statistics related to HIAs.

Most of the ICNs acknowledged that they do various tasks simultaneously; therefore, they do not have enough time for visiting wards for case finding. In Iran, regardless of the hospital beds, usually, there is an ICN in each hospital. So it can be an obstacle to proper case finding and accurate reporting of HAIs. This finding is in line with previous researches [16, 17, 19] which have shown lack of human resources is the main challenge of HAIs surveillance and IPC program. The suggested recommendations to solve this problem were calculating the number of required ICNs in each hospital according to the suggested formula in scientific evidence and omitting other unrelated tasks to the IPC program from the ICNs responsibilities. Several studies [13, 14, 20] recommended one ICN per 100 critical beds and one ICN per 150 to 250 long-term care beds. Zingg et al., based on a systematic review recommended a full-time ICN as the minimum standard for the IPC program per 250 beds [21].

Our study showed that an insufficient ratio of ICN to hospital beds can worsen HAI findings. Therefore, the number of ICNs required for a hospital should be determined based on the number of hospital beds and their potential workload.

The majority of participants also expressed that lack of laboratory equipment and funding are other issues that impact HAIs reporting in Iran. Rodriguez et al. and Moosazadeh et al., mentioned microbiological laboratory equipment is one of the important elements of HAIs control measure [9, 19]. The limitations of financial resources in the IPCs program also appear to be well supported by previous research [1, 17, 19, 22]. The expert panel suggested allocating an adequate budget to the IPCs program, providing appropriate equipment for diagnosis-related pathogens to HAIs to overcome these issues.

Worrying about a negative reputation and reducing the income of some physicians by hospitals or being introduced as the best hospital in HAIs reporting by MOH are two sides of a coin that contribute to inaccurate HAIs reporting by some of the participants. As previous researchers had highlighted [6, 23], it seems there is a significant connection between HAIs reporting and clinicians and hospital reputation and payment. Therefore, Centers for Disease Control and Prevention (CDC) insists on HAIs data validation because various incentives are for HAIs underreporting [20]. Carter et al. recommended measures be taken to ensure the confidentiality of collected data and develop an environment free from blame to encourage physicians and nurses in HAIs reporting [23]. The corrective recommendations that were suggested by the expert panel include defining indicators for clarification of the contribution of each part (staff, environment, and equipment) in HAIs development, assessing the performance of the healthcare team regarding IPC, providing feedback to them, applying encouragement and punishment measures to enhance collaboration of healthcare team in HAIs reporting. In conclusion, several factors contribute to stakeholders’ engagement in HAIs case finding and reporting. Therefore, responsible organizations should recognize the motivations of different stakeholders and take appropriate measures to address these motivations.

What is surprising is the fact that the changing of MOH national policy to encourage hospitals to improve their HAIs reporting resulted in some hospitals becoming more motivated to have a higher HIAs rate. Stone et al. noted that although financial incentives could be an important motivator for IPC programs, there is a paucity of evidence on the efficacy of these motivations on HAIs reporting [20]. To overcome these concerns, experts suggested using indicators of reporting process instead of reporting HAIs rates [20]. So, in evaluating IPCs programs, managers should not merely pay attention to the reported statistics. They should consider other factors such as how that program is implemented and the services which are provided by that hospital.

The participants experienced a low level of motivation when they have little expertise in the IPC program, were not paid adequately, were overwhelmed with various tasks, or did not hold an organizational position. Deficiencies in training were reported as one of the main challenges of HAIs surveillance [16]. This inadequacy of training leads to difficulties in HAIs case finding or diagnosis by IPC team members. In line with our finding, Mosazadeh et al., and Mahomed reported multitasking of IPC team members resulted in a lack of time for doing surveillance task [16, 19].

Then, the job motivation of the IPC team along with other factors could enhance case findings of HAIs [6]. Danchaivijitr also showed that a lack of organizational post for ICNs led to a lack of motivation among ICNs to implement IPCs programs. They reported that just less than one-fourth of hospitals have a full-time ICN, as a result, the ICN perform IPCs program as an in-training position. Therefore, a competent ICN does not remain in this position for a long time [1]. Welsh et al. reinforced the importance of continuous education and sharing the result of new research to enhance IPC performance [24]. The current study found that several factors influence on IPCs team members. It can thus be suggested that hospital administrators should be considered these influencing factors.

The elaborated responses expressed by participants indicated the importance of financial incentives for performing IPCs programs, especially HAIs case finding and reporting. Sok and Kanal (2013) reported that financial shortage is one of the barriers to the implementation of IPC programs in developing countries [8]. Mahomed expressed that providing incentives resulted in improving HAIs data collection and reporting [16]. The current studies confirm that financial support and monetary incentives can improve the implementation of IPC programs and the compliance of HCWs to implement IPC programs [25, 26]. The expert panel’s suggestion regarding inadequate motivation is in complete agreement with Rudasingwa and Uwizeye (2016) expressed that performance-based financing could improve the structures and process of health care [27].

This study was conducted in one medical university, the largest one in Iran, and with small sample size. The reader should bear in mind that generalizing findings from such qualitative work and single-site studies are based on logic or theory, not statistics and inferences. The study findings provide an important insight into the challenges and recommendations of HAIs case finding and reporting in a developing country. Then the result could be useful for other countries in same condition.

## Conclusion

The findings of this study add substantially to our understanding of challenges in HAIs case finding and reporting. The evidence highlighted that some drawbacks in resources and planning, conflicts of interest, and inadequate motivation of IPC team members lead to inaccuracy of HAIs reporting in the health system of Iran. The results demonstrate that accurate data in HAIs reporting is possible. The proposed corrective recommendations suggest several courses of action in order to solve these challenges at different levels such as hospitals, medical universities, and MOH. Future work should focus on implementing initiatives to enhance the quality of HAIs reporting in a health system.

## Data Availability

The datasets generated and/or analyzed during the current study are not publicly available due confidentiality of the participants but are available from the corresponding author upon reasonable request.

## Abbreviations

HAIs: Health care−associated infections
IPC: Infection Prevention and Control
NISS: Nosocomial Infection Surveillance System
ICNs: Infection Control Nurses
TUMS: Tehran University of Medical Sciences
INIS: Iranian nosocomial infections surveillance system
ICLNs: Infection Control Link Nurses
HIS: Hospital Information System
ICU: Intensive Care Unit
